# Sequential immune-targeted surgical therapy resulted in disease-free survival in a case with advanced renal cell carcinoma

**DOI:** 10.1186/s12894-021-00891-8

**Published:** 2021-09-08

**Authors:** Kenichi Nishimura, Noriyoshi Miura, Naoya Sugihara, Keisuke Funaki, Kanae Koyama, Yuichiro Sawada, Terutaka Noda, Tetsuya Fukumoto, Yuki Miyauchi, Tadahiko Kikugawa, Takashi Saika, Masafumi Matsumura, Katsuyoshi Hashine, Mashio Taniwaki

**Affiliations:** 1 Department of Urology, Ehime University, Shitsukawa, Toon, Ehime 791-0295 Japan; 2 Department of Urology, National Hospital Organization Shikoku Cancer Center, Minamiumemoto, Matsuyama, 791-0280 Japan; 3 Department of Pathology, Ehime University, Shitsukawa, Toon, 791-0295 Japan

**Keywords:** Sequential immune-targeted therapy, Advanced renal cell carcinoma, Nivolumab, Ipilimumab, Case report

## Abstract

**Background:**

Currently, immunotherapy is indicated for patients with metastatic RCC or unresectable RCC, but there is no indication for immunotherapy in the neoadjuvant setting. We report a case in which the combined use of nivolumab and ipilimumab and sequential TKI therapy enabled surgical treatment.

**Case presentation:**

A 71-year-old female was diagnosed with a metastatic clear-cell renal cell carcinoma with a level IV tumor thrombus. She was started on nivolumab-ipilimumab therapy, and was switched to pazopanib monotherapy because the tumor thrombus progressed within the right atrium. The tumor shrank to resectable status with sequential therapy. She then underwent right nephrectomy and thrombectomy. Pathological analysis showed 10–20% residual tumor in the primary tumor, but no viable cells in tumor thrombus. She remains clinically disease-free 1 year after surgery.

**Conclusion:**

This case suggests the utility of sequential immune-targeted therapy as neoadjuvant therapy in advanced renal cell carcinoma.

## Background

Renal cell carcinoma (RCC) has been reported to invade into the vena cava in 4–10% of cases [1]. Reese et al. reported the natural history of RCC patients with untreated tumor thrombus, and noted that 87% of these patients (n = 297) died of RCC with a median survival of 5 months [2]. In contrast, the 5-year survival rate after nephrectomy and tumor thrombectomy among RCC patients with tumor thrombus in the inferior vena cava (IVC) is approximately 50% [1]. Neoadjuvant therapy has been implemented in several cancer types to reduce the size of the tumor and improve surgical morbidity. However, currently no neoadjuvant systematic treatments exist for patients with advanced RCC. Multiple tyrosine kinase inhibitors (TKIs) have been evaluated in patients with locally advanced disease with the objective of downstaging to enable surgical resection. However, several studies have reported low rates of response [3]. Cost et al. reported that in 25 RCC patients with tumor thrombus, neoadjuvant TKI treatment led to a reduction of the thrombus in only 12% of patients and altered the surgical approach in only one patient [4].

Currently, immunotherapy is indicated for patients with metastatic RCC or unresectable RCC, but there are no indications for immunotherapy in the neoadjuvant setting. We report a case in which the combined use of neoadjuvant nivolumab and ipilimumab and sequential TKI therapy enabled surgical treatment.

## Case presentation

**Fig. 1:**
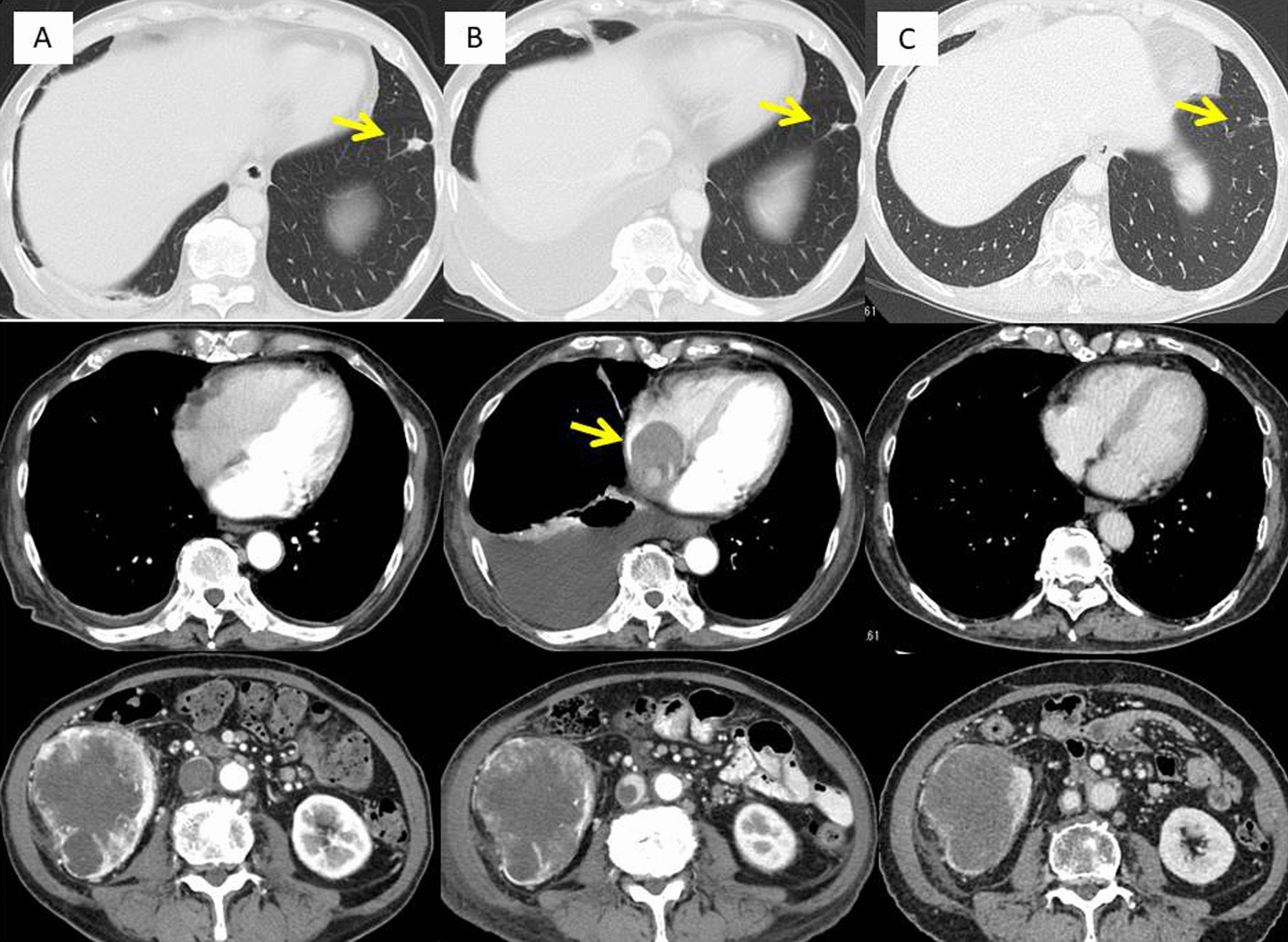
CT images showing lung metastasis and the primary tumor and tumor thrombus in the right atrium. **a** Before treatment. **b** After 2 cycles of nivolumab and ipilimumab. **c** Before surgery

**Fig. 2:**
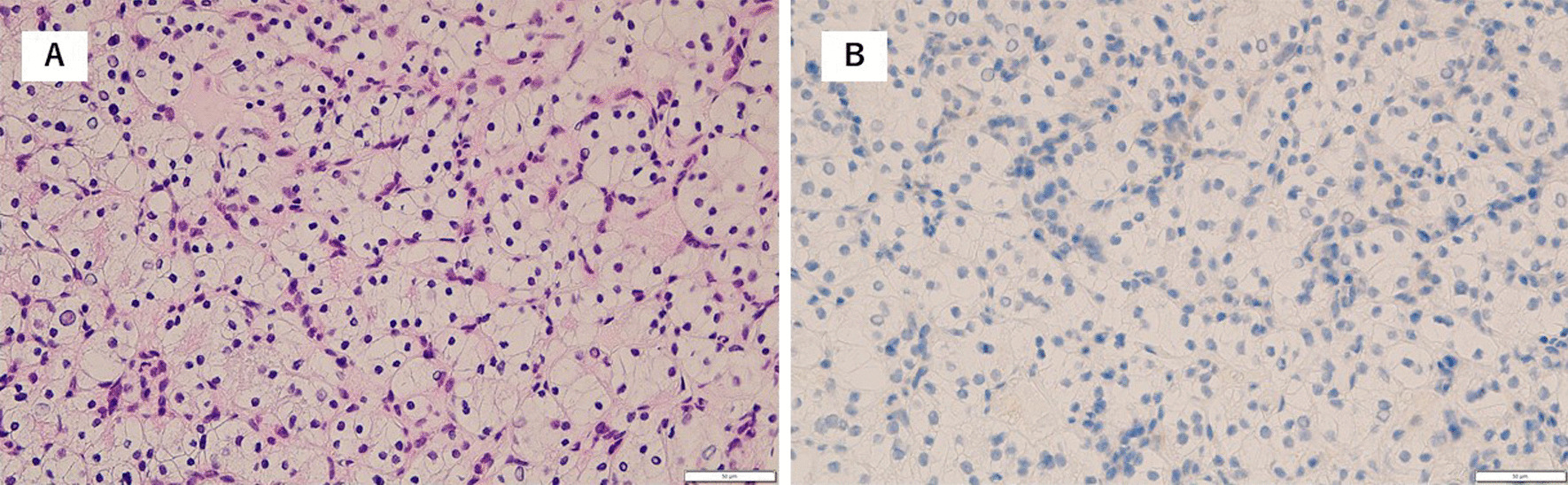
Pathological findings on needle biopsy. **a** Hematoxylin–eosin (HE) staining of the primary tumor (×40). **b** Immunohistochemical analysis of the primary tumor (×40)

A 71-year-old female presented with 8 kg weight loss over several months, appetite loss, and leg edema for several weeks. An enhanced computed tomography (CT) scan revealed a 94-mm right renal mass with a bulky tumor thrombus within the IVC to the junction of the IVC and the right atrium, maximum thrombus diameter of 37 mm, a few lung nodules, and para-aortic adenopathy (Fig. 1a). A transthoracic echocardiogram revealed no tumor within the right atrium. A bone scan revealed no metastasis. A core needle biopsy of the renal mass showed mostly necrotic tissue with a region of clear-cell RCC (ccRCC) (Fig. 2a). Immunohistochemical analysis revealed that PD-L1 was not expressed on tumor cells (Fig. 2b). The patient was not appropriate for radical surgery because her Karnofsky performance status (KPS) was 40. Systemic immunotherapy was administered for metastatic RCC based on International Metastatic RCC Database Consortium (IMDC) poor-risk classification including KPS < 80%, diagnosis to treatment interval < 1 year, anemia, and hyper calcemia. After 2 cycles of nivolumab and ipilimumab therapy, CT revealed that the primary tumor was stable at 94 mm in diameter and lung nodules were undetectable except for the one in the right lower lobe, but the tumor thrombus was extended within the right atrium. Nivolumab and ipilimumab therapy was changed to pazopanib monotherapy due to disease progression (Fig. 1b). She had marked improvement in Karnofsky performance status to 70 and resolution of leg edema and appetite loss.

Follow-up CT at 4 months after treatment revealed that the renal mass had decreased to 84 mm in diameter, and all lung nodules were undetectable. The tumor thrombus in the right atrium was also undetectable, but the tip of the thrombus remained at level 3. The diameter of the IVC at the renal vein ostium was 15.6 mm. Complete occlusion of the IVC was not observed (Figs. 1c, 4). She underwent right nephrectomy and IVC thrombectomy after 2 cycles of nivolumab and ipilimumab therapy and pazopanib therapy for 5 months.

The surgical method is described below. A cardiac surgeon secured the right upper arm vein and the right femoral vein to prepare for extracorporeal circulation. A middle incision to the xiphoid was made in addition to chevron incision. The right kidney and IVC were exposed. The area around the kidney and the IVC had strong adhesions. The right renal artery was ligated at the aortocaval. A tumor thrombus was confirmed between the common iliac vein bifurcation and the diaphragm by echoic imaging, but no tumor thrombus was observed in the right atrium. A liver surgeon secured the hepatic triad. Furthermore, an incision was made in the epicardium and the IVC was secured above the diaphragm. Since no change in blood pressure was observed when the IVC was clamped above the diaphragm, a decision was made to perform nephrectomy without using extracorporeal circulation. The hepatic triad was clamped using a Pringle, and the IVC (above the diaphragm and on the common iliac vein bifurcation) was clamped. The vascular wall was incised at the bifurcation of the renal vein and the IVC to secure the tumor thrombus. The tumor thrombus was manually removed from the IVC wall. However, dense adhesions were revealed between the tumor thrombus and the wall of the IVC. The IVC was resectable from beneath the hepatic vein to the IVC bifurcation. Unfortunately, the tip of the thrombus could not be removed. Since the tip of the thrombus was macroscopically firm necrotic tissue, no additional excision was performed. The operative time was 9 h, and the bleeding volume was 3000 mL. No surgical complications occurred. CT and a transthoracic echocardiogram 1 week after surgery revealed no metastatic findings and only the mass within the IVC near the right atrium. Treatment for RCC after surgery was not performed because we diagnosed the mass as having no viable cells by pathological findings. 1 year after surgery, the mass has not changed and no metastatic lesions have been observed.

**Fig. 3:**
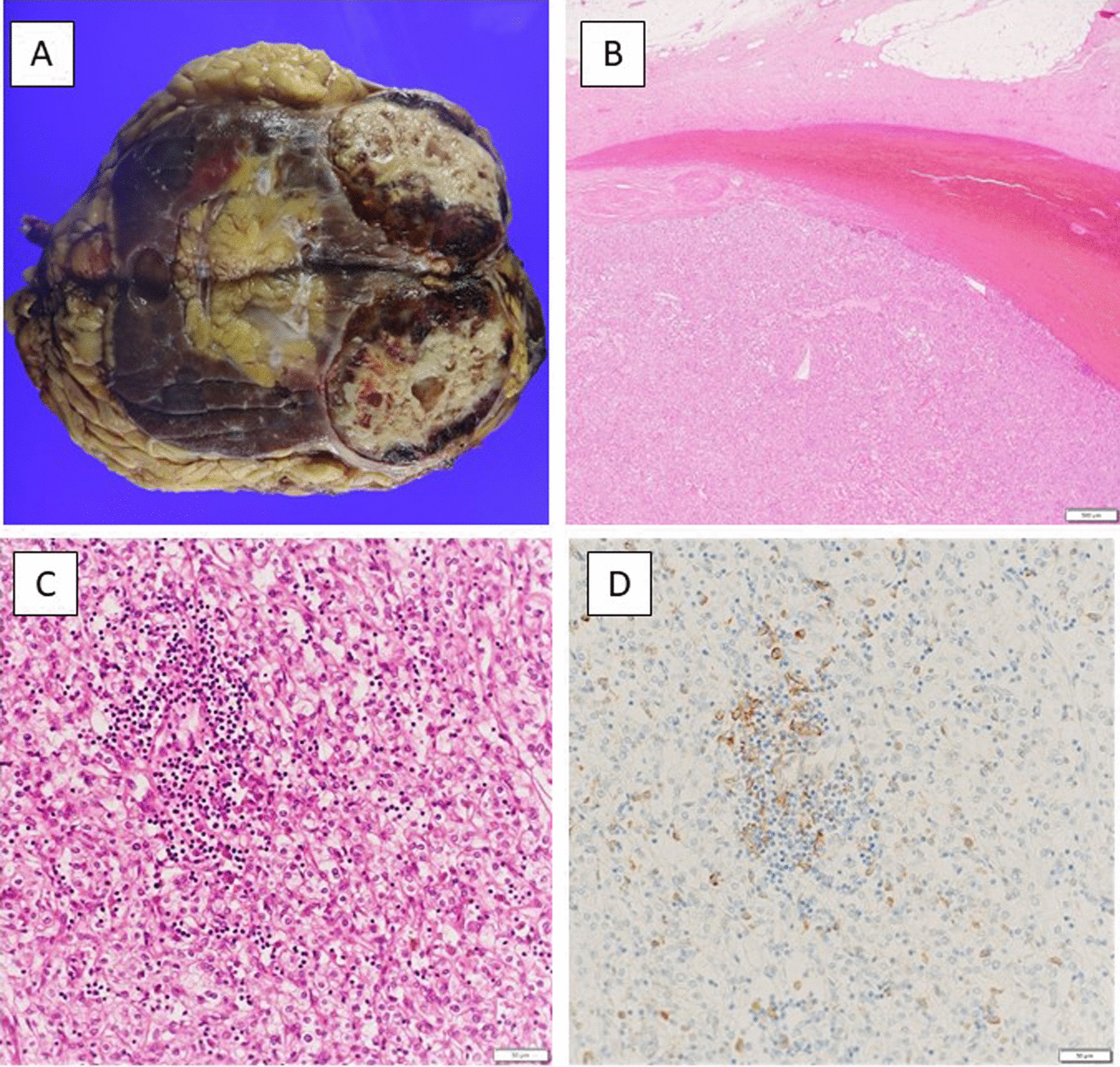
Pathological findings. **a** Macroscopic findings. **b** HE staining of the primary tumor (×2). **c** HE staining of the primary tumor (×40). **d** Immunohistochemical analysis of the primary tumor using 2 FDA-approved clinical immunohistochemical makers for PD-L1 expression: Dako PD-L1 22C3 pharmDx and Dako PD-L1 28-8 pharmDx (×40)

Finally, the pathologic diagnosis was ccRCC (7.5 cm, International Society of Urologic Pathologists [ISUP] Grade 3). Macroscopic findings showed a light yellowish-brown color in the margin area and extensive necrosis in the internal red and yellow areas (Fig. 3a). Histologically, tumor cells with clear cytoplasm and round nuclei were observed at the margin of the lesion. The proportion of residual tumor in the primary tumor was 10–20% (Fig. 3b, c). The tumor thrombus tissue was necrotic and replaced by a hemosiderin-phagocytic macrophage population. No viable cells were observed. No tumor was found in the wall of IVC. Immunohistochemical analysis revealed that PD-L1 was expressed on lymphocytes and macrophages infiltrating the tumor, but not on the tumor cells themselves (Fig. 3d).

## Discussion and conclusion

**Fig. 4:**
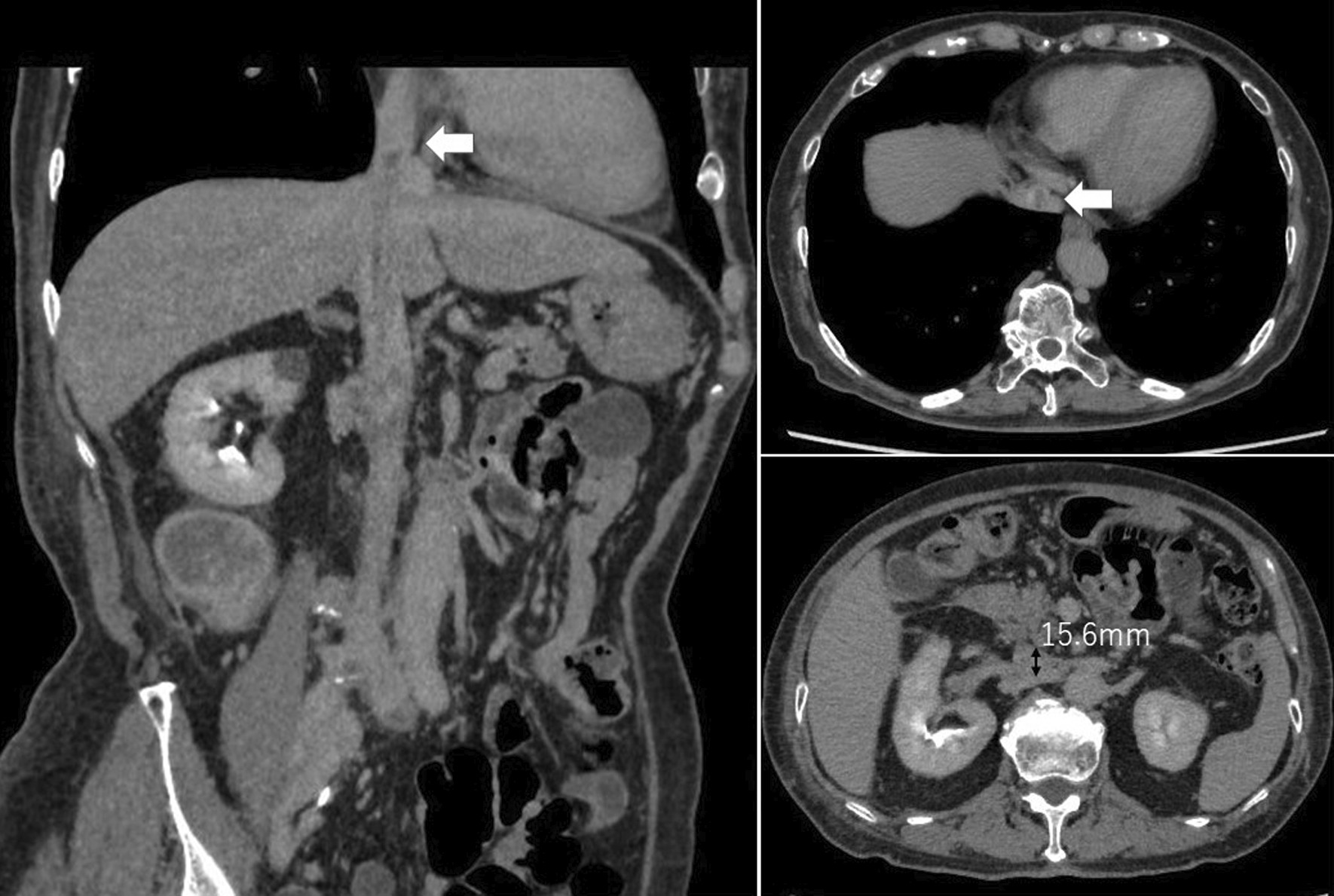
Characteristics of the preoperative tumor thrombus on enhanced CT. The white arrows indicate the tip of the tumor thrombus. The black arrows indicate the diameter of the IVC at the renal vein ostium (15.6 mm)

We have described a patient who initially presented with invasive RCC, a level IV vena cava thrombus, and multiple lung metastases. She initiated nivolumab and ipilimumab therapy, and was switched to pazopanib monotherapy upon disease progression. Then, a smaller tumor thrombus and lack of metastases were observed on enhanced CT. She underwent right nephrectomy and tumor thrombectomy. This is the first case report describing the utility of sequential immune-targeted therapy as neoadjuvant therapy in advanced RCC (Fig. 4).

At the beginning of her treatment, ICI + ICI therapy was covered by national health care insurance in Japan, but ICI + TKI therapy was not. Therefore, nivolumab and ipilimumab therapy was selected. 3 weeks after the first cycle of nivolumab and ipilimumab therapy, a tumor thrombus had progressed into the right atrium. We were forced to switch treatment due to an increased risk of sudden death. In patients with disease progression on immunotherapy, the possibility of pseudo-progression (PsPD) or hyper-progression must be considered. In PsPD, the tumor and/or metastatic lesions initially increase in size after immunotherapy due to infiltration of immune cells, followed by tumor shrinkage. The incidence of PsPD in RCC has been reported to be 2.9–8.8% [5, 6]. Currently, no biomarkers exist to distinguish PsPD from hyper-progression. Moreover, the use of biomarkers in patients treated with immune checkpoint inhibitors (ICIs) remains controversial and is under investigation. The neutrophil-to-lymphocyte ratio (NLR) is reported to be useful as a prognostic factor. Aly-Khan et al. investigated the utility of NLR in patients with metastatic RCC treated with ICIs. Decreases > 25 from baseline to 6 weeks after ICI therapy are associated with significantly improved outcomes in metastatic RCC patients. In addition, these authors reported that the NLR may be a helpful and available marker to diagnose PsPD [7]. In the present case, a 23% decrease in relative NLR was observed at 6 weeks after initiation of nivolumab and ipilimumab therapy (baseline NLR, 5.2; NLR at 6 weeks after treatment, [4]. No methods were available to diagnose PsPD at 3 weeks after the first cycle of nivolumab and ipilimumab therapy. We should consider the present results in the context of other relevant clinical details to assess the risk–benefit of continuing ICIs in each individual patient.

Our surgical findings demonstrated that the tumor thrombus and IVC were firmly adhered. Psutka et al. reported that the presence of a right-sided tumor, an IVC diameter at the renal vein ostium of ≥ 24.0 mm, and radiographic identification of complete occlusion of the IVC at the renal vein ostium are related to tumor invasion of the IVC wall [8]. In the present case, the presence of a right-side tumor criterion was met, so we predicted that the possibility of IVC invasion was low.

However, the tumor thrombus had to be removed with the IVC from the hepatic vein to the bifurcation of the IVC due to the firm adhesion. The tip of the thrombus could not be removed because it was firmly adhered to the IVC near the right atrium. Adhesion of the tumor and the IVC wall was assumed to be due to inflammation, because tumor cells were not observed in the wall of the IVC on pathological findings. Labbate et al. also reported dense adhesion of the tumor and surrounding tissue after neoadjuvant immunotherapy for RCC vena cava tumor thrombus [9]. In RCC patients treated with neoadjuvant immunotherapy, we suggest a careful surgical plan that includes the removal of surrounding organs due to adhesions.

On pathological findings, the tissue of the tumor thrombus was necrotic and replaced by a hemosiderin-phagocytic macrophage population. Shinagawa et al. also reported no viable cells in the tumor thrombus but some viable cells in the primary tumor after TKI and anti–PD-1 drug therapy [10]. This pathological analysis suggested that neoadjuvant therapy is more effective for tumor thrombi. Anti–PD-1 antibodies are bound to T cells for 20 weeks or longer after ICI administration [11]. By administering TKIs immediately after the second course of ICIs, the present case received immuno-targeted therapy by ICIs and TKI prior to surgery. Immuno-targeted therapy is currently reported to be useful for mRCC patients. However, its use has not been reported in the neoadjuvant setting.

The present case is valuable in that it suggests the utility of sequential immune-targeted therapy before surgery. We recommend that clinical trials of immune-targeted therapy in the neoadjuvant setting for RCC patients are conducted.

## Data Availability

All data and materials are available within the manuscript.

## Abbreviations

RCC: Renal cell carcinoma
IVC: Inferior vena cava
TKI: Tyrosine kinase inhibitor
CT: Computed tomography
ccRCC: Clear-cell renal cell carcinoma
PD-L1: Programmed death-ligand 1
IMDC: International Metastatic RCC Database Consortium
ISUP: International Society of Urologic Pathologists
PsPD: Pseudo-progression disease
ICIs: Immune checkpoint inhibitors
NLR: Neutrophil-to-lymphocyte ratio
